# On the possibility of using temperature to aid in thyroid nodule investigation

**DOI:** 10.1038/s41598-020-78047-1

**Published:** 2020-12-03

**Authors:** C. P. Damião, J. R. G. Montero, M. B. H. Moran, R. A. da Cruz Filho, C. A. P. Fontes, G. A. B. Lima, A. Conci

**Affiliations:** 1 Department of Internal Medicine, Federal Fluminense University, Rio de Janeiro, Brazil; 2 Computing Institute, Federal Fluminense University, Rio de Janeiro, Brazil; 3 Department of Radiology, Federal Fluminense University, Rio de Janeiro, Brazil

**Keywords:** Cancer, Endocrinology, Oncology, Mathematics and computing

## Abstract

Thyroid nodules are common, and their investigation is very important to exclude the possibility of cancer. The increase in blood vessels of malignant tumours may be related to local temperature augmentation detectable on the skin surface. The objective of this paper is to evaluate the feasibility of Infrared Thermography for cancer identification. For this purpose, two studies were performed. One used numerical modelling to simulate regional metabolic temperature propagation to evaluate whether a nodule is perceptible on the skin surface. A second study considered thyroid nodule identification by using convolutional neural networks (CNNs). First, variations in nodular size and fat thickness were investigated, showing that the fat layer has an important role in regional heat transfer. In the second study, the training process achieved accuracy of 96% for in-sample and 95% for validation. In the testing phase, 92% accuracy, 100% precision and 80% recall were achieved. Thus, the presented studies suggest the feasibility of using Infrared Thermography with the CNN Artificial Intelligence technique as additional information in the investigation of thyroid nodules for patients without a very thick subcutaneous fat layer.

## Introduction

Thyroid nodules (TN) are common in the general population. According to population studies conducted with adults living in iodine sufficient areas, approximately 4–7% of women and 1% of men have palpable thyroid nodules^1^. However, the prevalence of nodules estimated by ultrasound examination is higher, reaching up to 68% of the population^2^.

Thyroid Nodules represent an increase in thyroid volume with overgrowth and structural or functional transformations of one or more areas of the thyroid gland^3^. The factors that increase the chance of developing thyroid cancer are history of childhood head and neck radiation therapy or ionizing radiation exposure; history of thyroid carcinoma in the family or in a first-degree relative; and rapid nodule growth or hoarseness^4^. Although the majority of TN are benign, malignancy exclusion is mandatory. Currently, fine needle aspiration biopsy (FNAB) is considered the gold standard diagnostic tool for TN. Despite specificity greater than 95%, indeterminate FNAB results occur in 15–30% of cases^5^ and put both patient and surgeon in a treatment dilemma. Therefore, the development of other preoperative diagnostic techniques in these cases may avoid unnecessary diagnostic surgery. In line with this problem, Infrared Thermography has emerged as a low-cost and noninvasive method for investigating TN^6,7^. However, few studies have seriously evaluated this approach specially going up to the use of new Artificial Intelligence techniques, as the convolutional neural networks (CNNs), to promote a final conclusion on this subject as the here presented one^8^.

Thyroid cancer represents 7–15% of all TN^10^. Angiogenesis, the formation of new blood vessels, plays a pivotal role in the development and progression of thyroid tumours^12,13^. The increase in vascularity may be accompanied by an increase in local temperature. Furthermore, the increase in nitric oxide levels produced by malignant cell proliferation results in local vasodilation, which subsequently leads to heat emission^14^. Considering this information and the fact that the thyroid is a superficial gland^15^, the hypothesis that Infrared Thermography may be useful in the detection of malignant thyroid nodules is almost natural.

Among studies on the investigation of thyroid nodules by thermography, there was a report^16^ in 1972 of three nodules with histological diagnosis of malignancy. Two of them were described as “warm” on examination; however, the temperature of these tumours was not described^16^. In 1974, Galli et al. reported six cases of histologically confirmed malignant tumours that demonstrated an evidently increased cutaneous temperature by thermogram with values that were close to those recorded for hyperthyroidism; nevertheless, again, the temperature is not available^17^. In 1982, using a dynamic AGA thermovision (model 680) telethermograph, Di Pietro et al. evaluated 95 thyroid nodules that were operated on and concluded that there was no correlation between the thermal gradient and the diagnosis of malignancy^18^. Recently, Alves et al. performed a thermographic study with a digital (dynamic) thermography image and reported that 31 nodules suspected by thermography had malignancy confirmed in the intraoperative freezing examination, although the thermographic parameters were not described^8^.

In a previous study^19^, we used static thermographic examination data from eighteen (18) patients with malignant nodules; eighty-five (85) patients with benign nodules; and two (2) healthy patients (available in http://visual.ic.uff.br/thyroid/) to compare symmetrical neck parts. In this work, the minimum, maximum, average and median temperatures as well as the thermographic index^11^ and asymmetry parameter ($P_A$)^20^ were used to verify if the groups of patients could be differentiated by them in any combination. We calculated and tested hypotheses using two statistical tests: effect size^21^ and Wilcoxon–Mann–Whitney test^22^ with a confidence level of 95%. The results showed that the unique feature that can be consider is the $P_A$. For this reason this feature is used since then to test possible nodule location in our studies.

Considering the development of numerical models, the study by Conceição et al. consisted of heat transfer analysis in two three-dimensional (3D) geometrical models of the frontal cervical region around the thyroid gland and whether it contained a tumour^23^. An experimental study by Bahramian and Mojra investigated the feasibility of using thermography in conjunction with artificial neural networks for the detection of thyroid tumours. A 3D model of a healthy human neck was constructed based on computed tomography images, and this model was used to analyse the bioheat transfer in the human neck. Dynamic thermal images were captured (following the international recommendations) from two groups, one with 10 healthy patients and three thyroid cancer cases showing a significant variation in heat measured between these two groups^24^.

Machine learning (ML) algorithms have shown interesting applicability in medical-related problems^25^. Deep-learning methods, more specifically convolutional neural networks (CNNs), have achieved impressive results in medical image classification problems^25^ and can be investigated to help identify patterns in temperature maps related to the malignancy of thyroid nodules.

The main objective of the present study is to quantitatively evaluate the feasibility of using temperature for thyroid nodule identification. For this purpose, two studies are performed. In the first one, numerical models for thyroid simulation are developed to represent the metabolic process of temperature propagation and evaluate how much the presence of a nodule is perceptible through the temperature of external tissues, considering the nodular size and the presence of surrounding insulating tissues. Therefore, a theoretical assessment of whether the temperature of the thyroid surface can actually be used in the nodule identification process is needed. In the second study, the feasibility of using thermographic images in the identification of thyroid nodules with the help of image processing techniques, pattern recognition, and CNN is evaluated. In this way, from the union of both studies, this work addresses the problem of usability; that is, when the use of temperature measured by infrared cameras can help to identify the thyroid nodule malignancy.

## Results

### Study 1: bioheat transfer analysis

This study presents the thyroid numerical models developed to simulate how temperature behaves in the thyroid region and how anatomical characteristics determine the temperature transition to skin.

#### Variation in temperatures inside the neck

**Figure 1:**
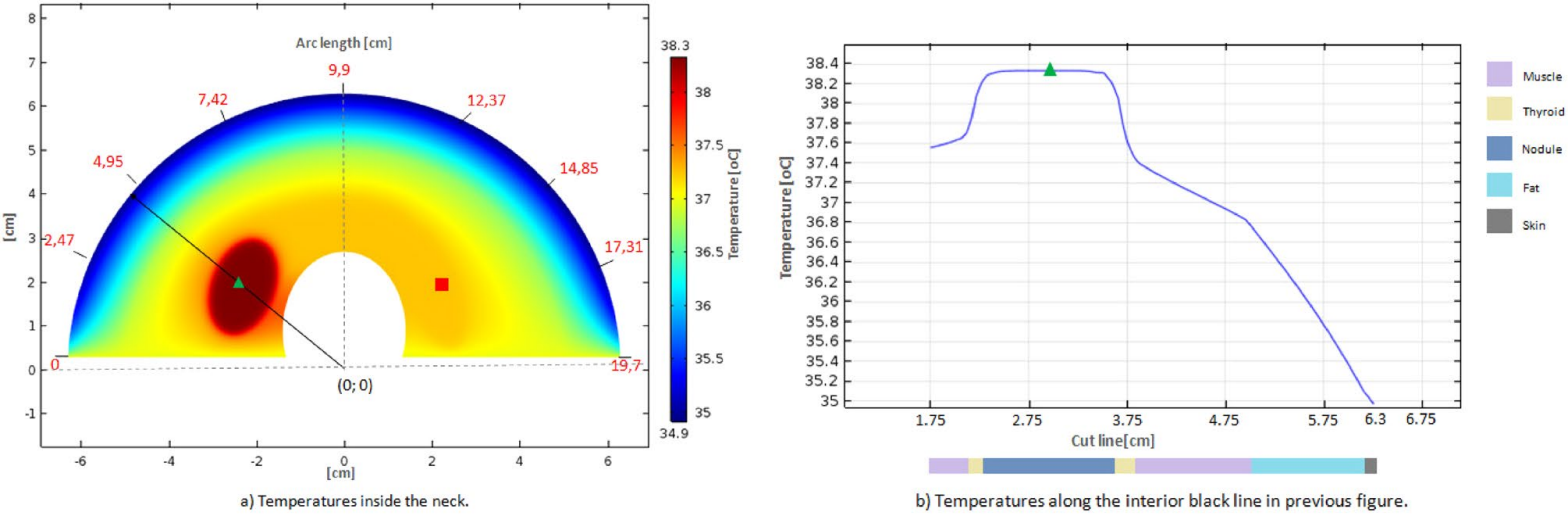
Temperature variations by the numerical simulation for elliptic malignant nodules of 1.0 $\times$ 1.57 cm and fat tissue layer thickness of 1.2 cm. (**a**) Temperatures on the cross section and along the skin surface of the neck, where the red number indicated the position on skin (i.e. arc length) beginning in the left end of the section. (**b**) Temperatures along the black line (semi circle radius) and center of the nodules (green triangle) showed in (**a**), the length (showed in the horizontal axis) begging in the center of the circle section.

Figure 1a shows the temperature variations in the numerical simulation when considering a thickness of 1.2 cm for the fat tissue layer and elliptic malignant nodule with diameters of 1.0 $\times$ 1.57 cm. Temperature values of approximately $37.2 \,^{\circ }\hbox {C}$ were obtained in the right thyroid lobe region (in the red square position), the region without a nodule, which corresponds to normal body internal temperature. Values of $38.4\, ^{\circ }\hbox {C}$ were found in the central region of the nodule (in the green triangle position), which is the same as that shown in a previous study^23^. The red numbers in Fig. 1a indicate the longitude of the arc length at the skin surface, which measures approximately 19.7 cm when a thickness equal to 1.2 cm of the fat tissue layer is considered. No differences in temperature were found on the neck surface, i.e., the neck surface along the arc length or comparing the contralateral region of the nodular area. Figure 1b shows the temperature variations in the numerical simulation along a line crossing the nodule from the trachea to the skin. The temperature variation in the muscle layer (from the nodule to the skin, i.e., on the right of the nodule layer of Fig. 1b) was approximately equal to $0.5\,^{\circ }\hbox {C}$ (from 37.4 to $36.9\,^{\circ }\hbox {C}$), while the temperature variation in the fat layer was $1.8\,^{\circ }\hbox {C}$ (from 36.9 to $35.1\,^{\circ }\hbox {C}$).

#### Analysis of the influence of the size of nodule and fat tissue thickness

**Figure 2:**
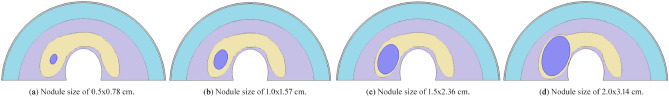
Considered variations in the nodule size.

Four size of nodules are considered: 0.5 $\times$ 0.78 cm, 1.0 $\times$ 1.57 cm, 1.5 $\times$ 2.36 cm, and 2.0 $\times$ 3.14 cm, as shown in Fig. 2. Moreover, various fat thicknesses are considered because neck surface temperature may be related to the thickness of the fat layer due to the insulation effect of the adipose tissue. The fat tissue layer varies among individuals, its thickness is related to the body mass index and can be measured by ultrasound (US) examinations^26^.

**Figure 3:**
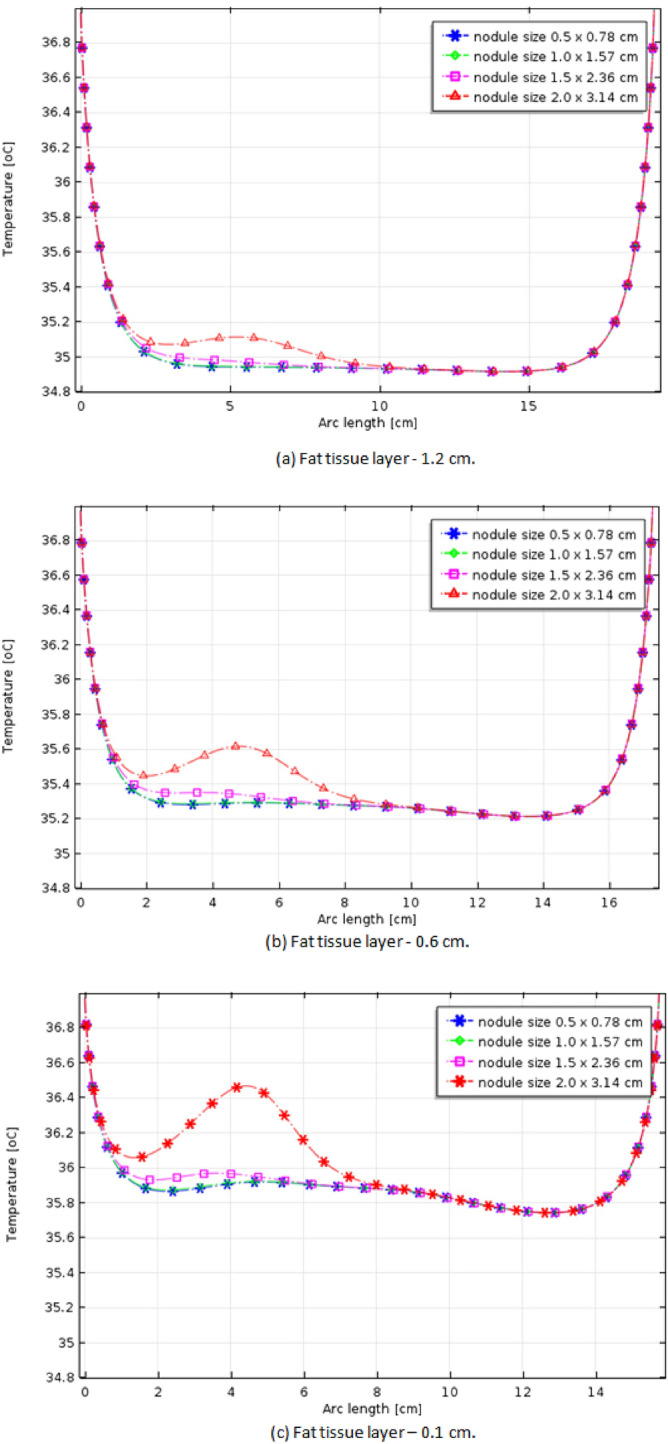
Thermal profiles on the neck surface for the four tumour sizes in Fig. 2 and the three fat tissue layers.

The curves in Fig. 3 show the thermal profiles obtained from the simulations of malignant nodules of Fig. 2 on necks with 1.2 cm (Fig. 3a), 0.6 cm (Fig. 3b) and 0.1 cm (Fig. 3c) fat tissue layer thickness. As seen, there is a perceptible surface temperature variation just in front of the nodule (approximately 4.2 cm in the fourth graph) when compared to the contralateral region (approximately 12.8 cm) for the smaller fat layer considered.

### Study 2: measurements of skin temperatures

In this study, we evaluated thermographic images to analyse whether nodules can be identified in these images, considering the superficial skin temperatures measured by the infrared camera.

For this study, thermographies of 25 patients with nodules are considered. A pre-processing step extracts 109 regions that present abnormal temperature behaviour. Twenty-six (26) of those regions are actually nodule related, and the remaining (83) are not nodule related. Different CNN models are considered for the classification of these images as nodule related or not. Twenty-five (25) of the 109 regions are separated to be used in the testing phase, and the remaining (84) are used in the training phase. Sixteen (16) of the 84 regions are nodule related, and the remaining (68) are not nodule related. Due to the difference in the number of elements for each class, two data augmentation processes^27^ (detailed in the “Methods” section) are performed, one for the nodular regions and the other for non-nodular regions, generating 528 images, which are used for model training.

The algorithms used in this study are based on the ResNet^28^ architecture, which has previously achieved excellent results for classification problems involving medical images^25,29^. The models used in this work were previously pre-trained using the ImageNet dataset^30^ to achieve better initial weight values. The fine-tuning training process included 2000 steps. Three values (0.1, 0.01 and 0.001) were used as the initial learning rate to evaluate which of these parameters would be the most appropriate. Thus, three ResNet models were considered, one for each defined learning rate variation. A cross-validation approach was not used due to the limitations in the available hardware environment. Table 1 shows the values of accuracy and loss obtained for in-sample and validation after the training process for the three learning rates. As observed in Table 1, the model that presented the best results during the training process used 0.1 as the learning rate.

**Table 1:** Results of the training process of each model in percentage.

Learning rate	Accuracy		Loss	
	In-sample	Validation	In-sample	Validation
0.1	96	95	14	8
0.01	94	90	16	29
0.001	92	87	18	25

Therefore, the model that used 0.1 as the learning rate was evaluated considering the 25 regions that were originally separated for testing. The statistical measures used in the model evaluation are true negatives (correctly classified negative examples), true positives (correctly classified positive examples), false negatives (positive examples incorrectly classified as negative), and false positives (negative examples incorrectly classified as positive)^31^. Such values are in the confusion matrix presented in Table 2, where it is possible to see that the total of examples correctly classified (accuracy) is 92%.

**Table 2:** Confusion matrix of the selected model.

	Predicted	
	Nodular	Not nodular
**Actual**		
Nodular	32%	8%
Not nodular	0	60%

**Figure 4:**
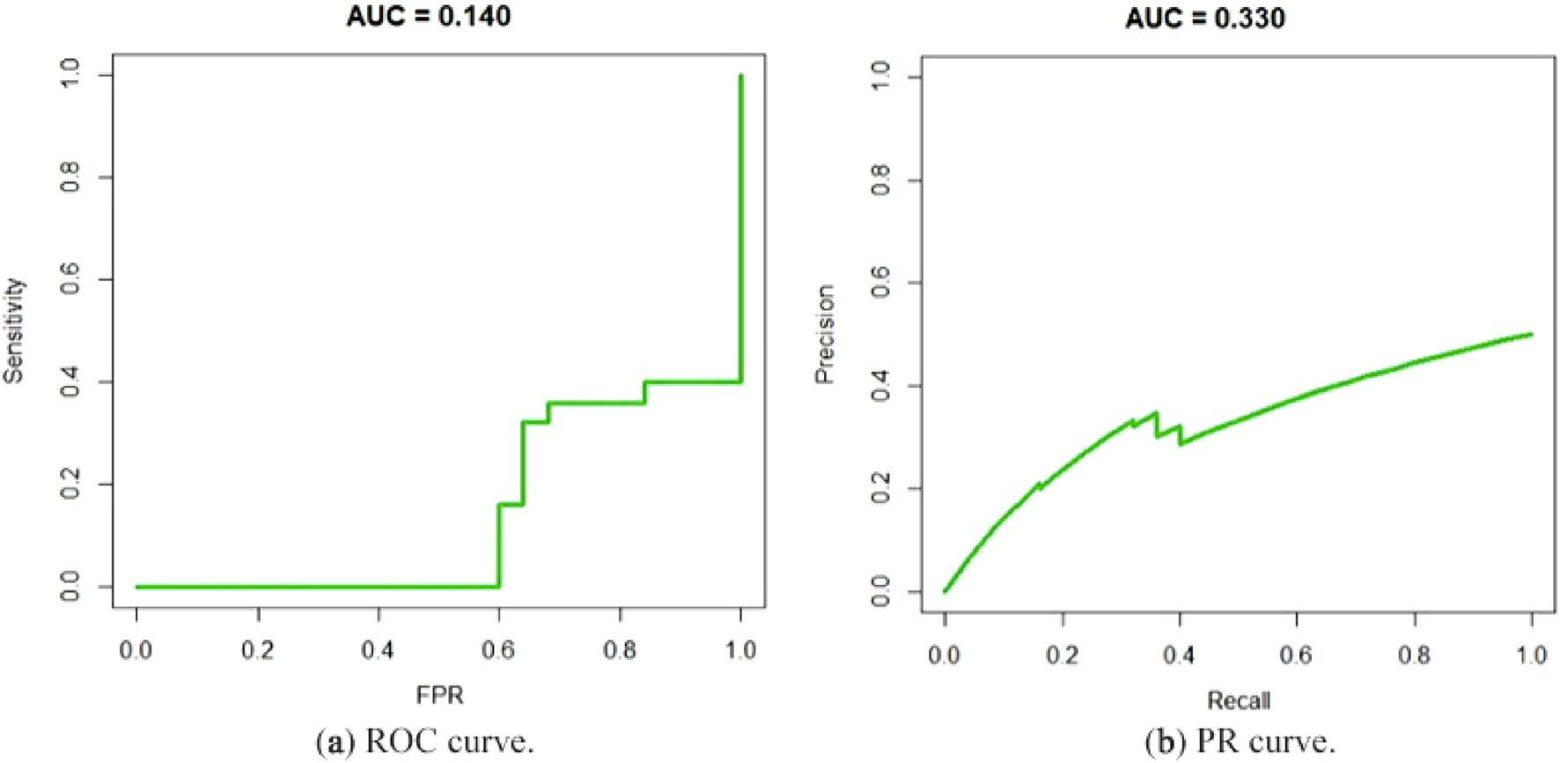
CNN used model performance.

The recall and precision were computed to show the model’s performance. Precision is the fraction of true positives among the sum of true positives and false positives. Recall is the fraction of true positives among the sum of true positives and false negatives. As observed in Table 3, the values found for precision and recall are 100% and 80%, respectively. The receiver operating characteristic (ROC) and precision and recall (PR) curves are shown in Fig. 4. They are fundamental for understanding and measuring relevance^31^. They present values of the area under the curve (AUC) of 0.14 and 0.33 (Table 3).

**Table 3:** Model performance on the test dataset.

Precision	Recall	AUC-ROC	AUC-PR
1.0	0.8	0.14	0.33

## General discussion

The results of Study 1 show that heat transfer for the neck with malignant thyroid nodules depends on the nodule size and mainly on the thickness of the fat layer. That is, the results demonstrated that fat tissue has an important insulating effect and should be considered in modelling the heat transfer from the TN to the neck skin.

The results of Study 2 suggest that nodule temperature, represented by thermographic images, may indeed indicate the presence of thyroid nodular regions. This is initially noted by the accuracy achieved during the algorithm training process. Evaluating the results for the test set, it is possible to observe that the classification of possibly nodular regions is performed impressively, since 92% (32 + 60) of all cases were correctly classified, as seen in the confusion matrix in Table 2. Thus, if we analyse the classification as a binary decision task, the result presented by the CNN is considered adequate. However, analysing the probabilities for each class assigned in each evaluated case, which can be observed generally in the ROC and PR curves (Fig. 4), it appears that the model cannot unequivocally distinguish the classes. The AUC for both curves can be considered low, which identifies the weakness in the developed model.

Considering the results of both studies, there is evidence that temperature is an information to be considered; however, this is restricted to patients with a relatively thin fat layer. This was evident in the results of Study 1 and suggested by cases of success and error of the method proposed in Study 2. The value of 8% in Table 2 represents two cases in which the algorithm incorrectly classified a nodular region as non-nodular. These nodules are from the same patient: a female with degree II obesity (i.e., mass of 85 kg, 1.54 m tall and body mass index—BMI—of 35.8 kg/m$^2$), and the nodules are relatively small (2.0 $\times$ 0.8 $\times$ 1.0 cm and 2.0 $\times$ 1.0 $\times$ 0.6 cm). Thus, there is a perspective for the applicability of temperature to the identification of thyroid nodules. As future studies, it is very important to properly evaluate the neck fat layer of the patient before any consideration of the feasibility of using Infrared Thermography exams for TN investigation. For instance, this can be done by US thickness measurements of subcutaneous adipose tissue layer in the same US exam used to nodule size evaluation^26^.

## Methods

This project was approved by the Research Ethics Committee of the Federal Fluminense University Medical School—CEP CMM/HUAP no 1.776.071. Patients were included in the study after previously receiving an explanation about the research, agreed to use their data and sign the informed consent form. All exams of the patients used in this work (for instance Bethesda, Chammas, TSH, histopathology nodule details, patient history, height and weight—for BMI computation), as well as their images acquired following the international recommendations (as described in previous work^9^) are available under request.

### Study 1: bioheat transfer analysis

This section shows an analysis of the influence of the neck fat layer thickness and the size of a thyroid malignant nodule on heat distribution on the surface of the neck skin. For these purposes, Pennes’s bioheat transfer model^32^ was used. The various thermophysical properties linked to the metabolic heat generation of the tumour, the thermophysical properties of the tissues involved, and the various boundary and initial conditions are included in this heat transfer problem. The simulations used finite element analysis by the Comsol Multiphysics software version 5.2 , licence number: 1042008.

#### Mathematical model

Heat transfer analysis often considers transient time and spatial variation thermal exchange, both on the surface of the skin and within biological organisms. This analysis also considers variations in blood flow rate, vascular architecture, and thermal properties^33^. Pennes considers the total energy balance and its storage, internal energy rate, heat conduction, convection inside and outside the body and environment, as well as local heat generation. Chemical and electrical effects are not considered in this equation. The body was approximated by an homogeneous solid biological medium with isotropic thermal properties. The energy balance assumes that blood flow within the tissue is nondirectional at the capillary level, i.e., the capillaries are assumed to be oriented concerning their arterial and venous connections. Pennes’s equation Eq. (1) consists of a modified transient heat conduction equation and two heat sources, both per unit of time and volume: a heat source due to the metabolic effect $Q_m$ and a heat source due to the energy exchange between tissue and blood $Q_{b/t}$. In addition, this equation considers a source of external heat $Q_e$, which is not included in the present study.

1 $$\begin{aligned} pc\frac{\delta T}{\delta t} = k \nabla ^{\wedge } 2 T + Q_{b/t} + Q_m + Q_e \end{aligned}$$

where *p*, *c* and *k* represent the specific mass, specific heat, and thermal conductivity of tissue, respectively. The $Q_{b/t}$ heat source Eq. (2) depends on blood perfusion $w_b$ (i.e., volumetric blood flow rate per unit volume of tissue), the volumetric mass $\rho _b$ (or density) and the specific heat $c_b$ of the blood, and the arterial blood temperature $T_b$ at the capillary level.

2 $$\begin{aligned} Q_{b/t} = w_b \rho _c c_b (T_b - T) \end{aligned}$$

The $Q_{b/t}$ heat source is characterized by the convective heat transfer effectuated by the blood through the capillary vascularization present in living tissues, which is proportional to the temperature difference of the arterial blood entering the tissue and the temperature of the venous blood coming out of the tissue^34^.

#### Geometry and thermophysical parameters

**Figure 5:**
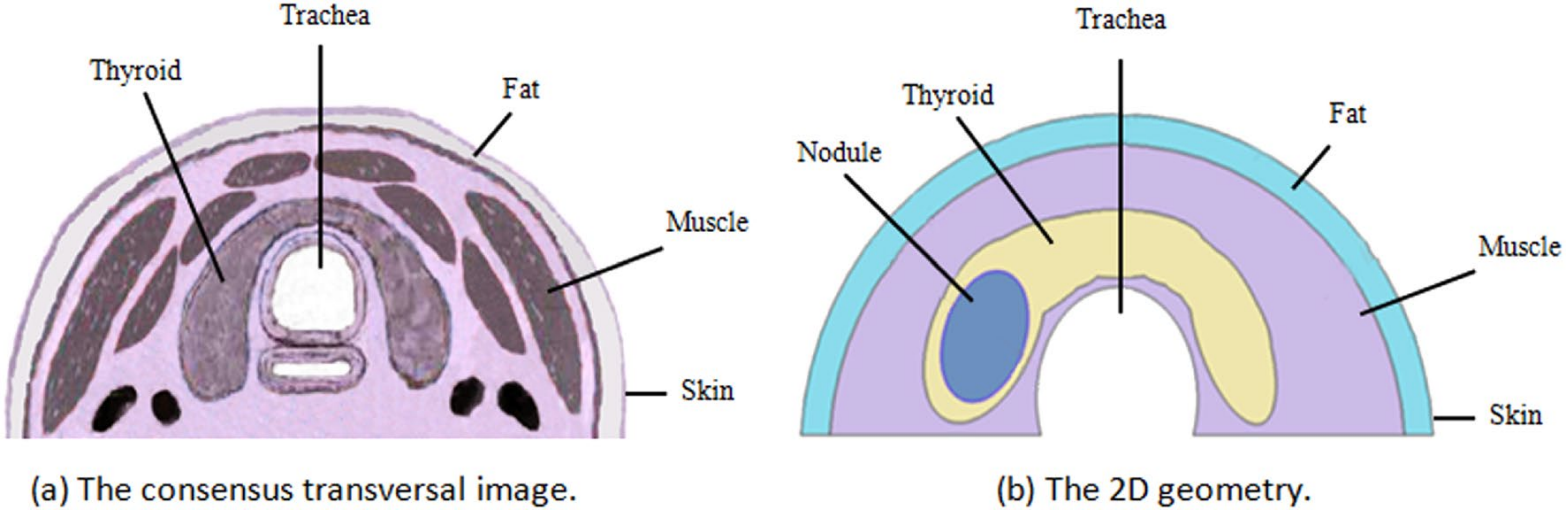
Relevant elements of the neck region in the best view of the thyroid gland and used numerical finite element model.

Based on the consensus image of an average human neck shown in Fig. 5a, the 2D simplified geometry of the cross section on Fig. 5b is used. This consensus image corresponds to a transverse section of the neck in the best view of the region, where entire thyroid gland can be represented in the transverse plane (CC axis)^35^. The simulated neck considers the main region components: skin, fat, muscle (and fascia) layers and thyroid gland with an elliptic malignant nodule inside it. The thicknesses of the skin and muscle tissue layers are 0.2 cm and 1.0 cm, respectively in Fig. 5b. The thickness of the fat layer and the nodule size vary in the simulation of this work for specific objectives. Primitive geometric elements (cylinders and ellipses) plus logical operations (union and differential), available in the Comsol Multiphysics software, were used to create the geometry of the model.

Table 4 presents the values of thermophysical parameters for each tissue. The $\rho _b$ and $c_b$ parameters are considered equal to the specific mass ($\rho$) and specific heat (*c*) of the respective tissue.

**Table 4:** Thermophysical parameters for tissues.

Parameter	Symbol	Skin	Fat	Muscle	Thyroid	Nodule	Units
Thermal conductivity	*k*	0.37	0.21	0.49	0.52	0.89	W/($\hbox {m}\,\hbox {K}$)
Specific mass	$\rho$	1109	911	1090	1050	1050	$\hbox {kg/m}^{3}$
Specific heat	*c*	3391	2348	3421	3609	3770	J/($\hbox {kg}\,\hbox {K}$)
Blood art. temp.	$T_b$	37.0	37.0	37.0	37.0	37.0	$^{\circ }\hbox {C}$
Blood perfusion	$w_b$	0.00196	0.000501	0.000708	0.098	0.465	1/s
Metabolic heat	$Q_{met}$	1829.85	464.61	1046	91455	2455386.6	$\hbox {W/m}^{3}$

#### Boundary and initial conditions

The simulations start from the initial conditions of air temperature of $20\,^{\circ }\hbox {C}$ . Thermal insulation was considered in the trachea surrounding region. On the remaining surfaces the temperature was assumed as $37\,^{\circ }\hbox {C}$ , even on the boundary at perpendicular axis that correspond to the internal human body temperature. At the skin surface, thermal convection with the environmental air had a convection coefficient of 3.6 W/($\hbox {m}^{2}\,\, {}^{\circ }\hbox {C}$).

### Study 2: identification of thyroid nodules by thermography

The thyroid anatomy is very symmetrical horizontally, considering the cranial-caudal axis. It is also a superficial organ that may allow the detection of heat caused by the hypermetabolism of nodules. However, there are thyroid regions that are normally highly vascularized and therefore present higher temperatures. Such regions are usually present on both sides of the thyroid, almost symmetrically. If symmetry is considered in the temperature evaluation, normal variations due to the regional anatomy do not affect the results^20^. The asymmetry parameter ($P_A$) is defined by the temperature difference of a region relative to its contralateral side^20^. Its values above $0.3\,^{\circ }\hbox {C}$ can indicate a dysfunction, and in general, if the value exceeds $1^{\circ }\hbox {C}$, a more significant dysfunction may occur in the region^20^.

**Figure 6:**
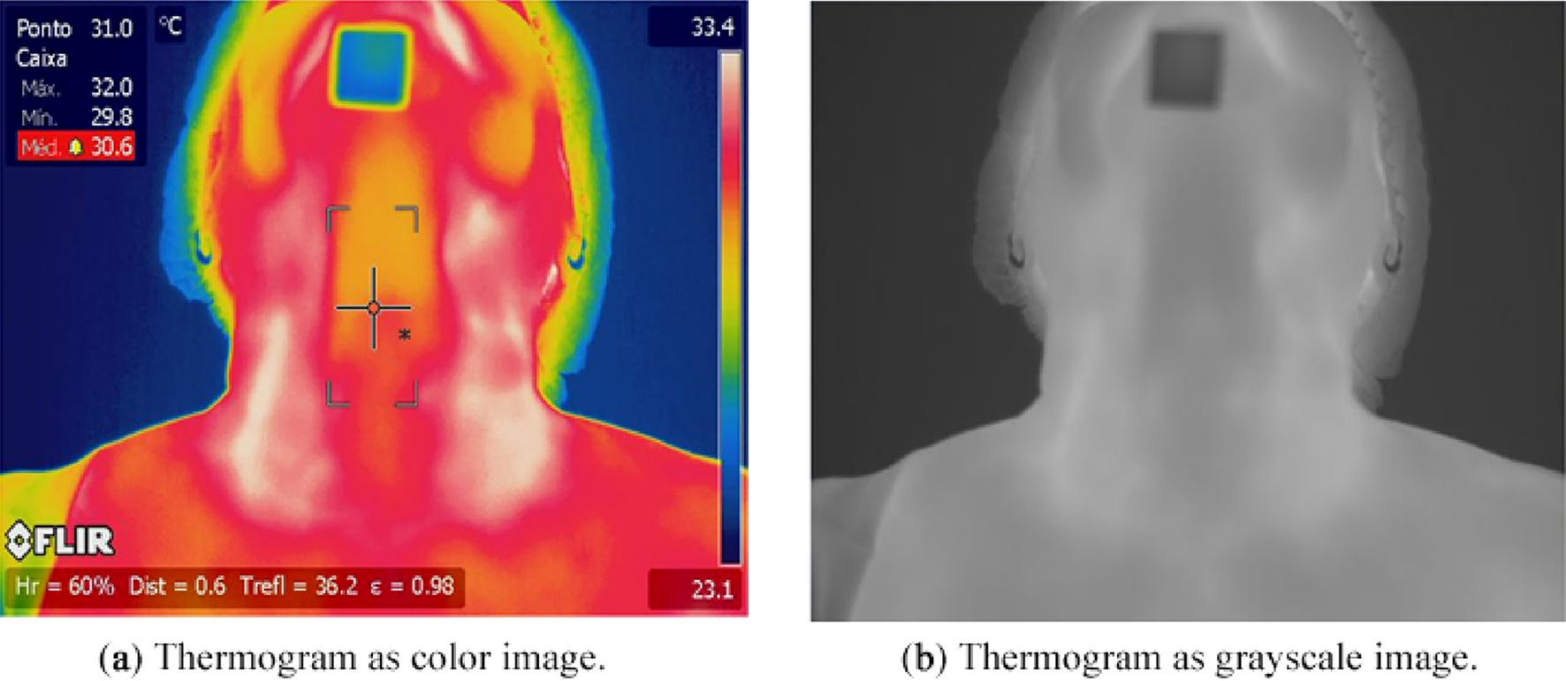
Thyroid thermography.

Thermographic images show the discrete temperature distribution of the scene in the frame captured by the camera at the acquisition time using the international protocol^9^ . This temperature distribution is represented by integer numbers as image *I* (Fig. 6). The number of pixels of the *I* image is defined by the camera resolution and consequently by the number of infrared sensors of the camera model used. In the case of the camera model used in this research (FLIR T620sc), the resulting images have a resolution of 640 $\times$ 480. Such a generic image *I* can be represented by the function $I (x, y) = i$, being a false colour (Fig. 6a) or a grey level (Fig. 6b) that represents a temperature value^19,36,37^.

From the frame captured by the camera, a bi dimensional matrix of temperature composed of real numbers *M* (640 $\times$ 480) can be obtained using a tool developed in previous works of the group^38^. This matrix can be represented by the function $M (x, y) = m$, which returns a temperature value in degrees Celsius given a position (*x*, *y*), where *x* $\in$ [1, 640] and *y* $\in$ [1, 480]. In other words, both *I* and *M* have the same number of elements^19,36^.

**Figure 7:**
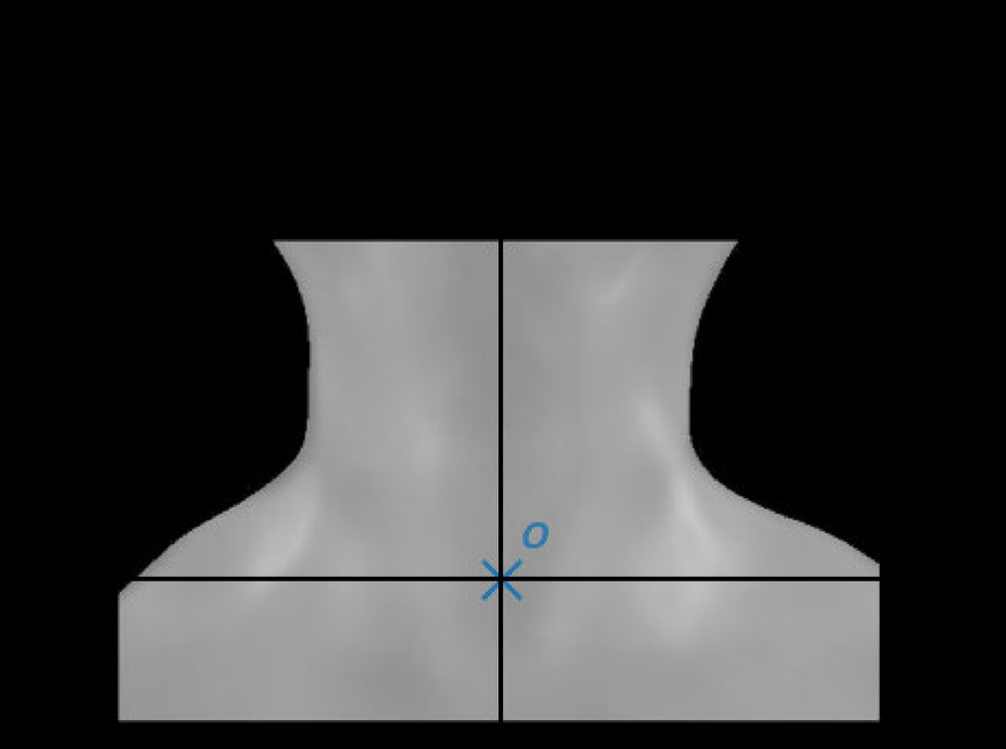
ROI (all rectangular area), $ROI_p$ (points different of black), $ROI_p$’s geometric centre *O* and, its vertical and horizontal axis, defined by $(o_x, o_y)$.

*I* and *M* are processed to define a region of interest (ROI) that considers only the area of the patient’s neck (Fig. 7). In the ROI image, the points have their values mapped to [1, 255] in a grey-scale range, and the other points have their intensity values defined as 0, becoming part of the background region (in black colour).

For practical purposes, in this work, the term $ROI_p$ refers to the set of points belonging to the ROI area, disregarding the background (black area). That is, $ROI_p$ consists of the set of points for which $I (x, y) \ne 0$.

Considering the $ROI_p$ of each patient, the vertical axis used to calculate the asymmetry parameter, $P_A$, was based on $ROI_p$ geometric centre *O*, defined in relation to the beginning of the *ROI*, i.e point (0, 0), by the coordinates $(o_x, o_y)$:

3 $$\begin{aligned} O = (o_x, o_y) = \left( \frac{\sum _{x=1}^{N_x} \sum _{y=1}^{N_y} x * ROI_p(x,y)}{N_p} , \,\, \frac{\sum _{x=1}^{N_x} \sum _{y=1}^{N_y} y * ROI_p(x,y)}{N_p}\right) \end{aligned}$$

where $N_x$ and $N_y$ are the $ROI_p$ limits and $N_p$ denotes the cardinality of the set $ROI_p$ (or the total number of pixels of the $ROI_p$). Therefore, the symmetric axis *e*(*y*) considered is defined by the horizontal coordinate of *O*, i.e. : $e(y) = o_x$

The $P_A$ of each point (*x*, *y*) $\in$ $ROI_p$ can be described as the difference of temperature between (*x*, *y*) and its contralateral $( (2 * o_x - x), y)$ position in relation to the *e*(*y*) axis on the same vertical height. Therefore $P_A$ of a point (*x*, *y*) is given by:

4 $$\begin{aligned} P_A (x,y) = M(x, y) - M((2 * o_x - x), y) \end{aligned}$$

where (*x*, *y*) is the coordinate any point $\in$ $ROI_p$. Note that the position of nodule candidate must be indicates by the hottest side. So, if the value of Eq. (4) is positive, nodule possible position is defined by the coordinates (*x*, *y*), because this is the hottest side. If the value of Eq. (4) is negative then $((2 * o_x - x),y)$ is the hottest point and $((2 * o_x - x),y)$ is the position to be considered as possible nodule. Only points belonging to $ROI_p$ are considered in this evaluation.

Thus, for each $ROI_p$, the vertical axis *e* is defined, and the $P_A$ values for that $ROI_p$ were calculated. After that, the following segmentation was performed: the $ROI_p$ points with $P_A$ above a threshold *l* were considered as belonging to a possibly nodular region. However, among these regions, there was still considerable noise. To eliminate part of this noise, the opening morphological operation was applied^19^. This operation consists of the combination of erosion and dilation operations. After that, the remaining regions were only those with more rounded shapes and larger dimensions, which have an aspect more similar to real nodules^19^. To obtain the images that are used as input to the CNN algorithms, new images are created by cropping the ROI considering the limits of the bounding boxes that cover each of the remaining regions^19^.

For each thermal exam, the result of this process was evaluated by a medical specialist, who compared the resultant regions with the data from ultrasound and other exams, indicating the regions that actually described nodules. This information was used as labels for CNN training and testing.

It should be noted that although these possibly abnormal regions have similar shapes, there are still some characteristics that can be used to classify them and indicate which regions refer to nodular regions. In this study, ResNet^28^ CNNs were used in the classification process (but AlexNet and GoogLeNet have been investigated in previous work^36^).

To be processed by the ResNet algorithms used here, the input images (crops of possibly abnormal areas) were resized to present the $224\times 224\times 3$ format, i.e., to have 224 lines, 224 columns and three channels (RGB). Moreover, the intensity values, which were initially in the [0, 255] range, were normalized to the [0, 1] range.

A data augmentation process was used to increase the training set because a large quantity of training data is essential for the success of CNN^31^. For this, we have applied transformation techniques in the initial image set to generate new data^31^. Thyroid nodules present a similar appearance in both (the right and left) lobes; therefore, the horizontal flip operation is a transformation that was used. The change in pixel intensity is another applicable transformation. However, the very used re-scale operations are not appropriate, since them modify nodule size with could change important information about the case in analysis. Consequently, the operations used in this work were only horizontal flipping and histogram stretching.

Finally, due to the difference in the number of elements in each class, we performed two different data augmentation processes. The process for non-nodular regions consisted of one horizontal flipping and one histogram stretching. The process for nodular regions consists of one horizontal flipping and three histogram stretching. Each operation doubles the number of elements.

In this work, after the data augmentation process, we obtained 256 images for the nodule class and 272 images for the non-nodule class.

### Ethical statement

The study was approved by the Research Ethics Committee of the Federal Fluminense University (CAAE, registered at the Brazilian Ministry of Health under project number 57078516.8.0000.5243), and all the methods applied were carried out in accordance with relevant guidelines and regulation. Informed consent was obtained from all persons considered in this research.

## Conclusions

Study 1 demonstrated that when using thermography for thyroid nodule identification, the thickness of the fat layer should be considered due to its insulating effect (and because it did not promoted difference measurable by the used camera in case of fat layer of 0.6 cm or more).

The results of Study 2 suggested that nodule temperature, defined from difference of temperature between points symmetrically located in relation to the neck vertical center and represented as visual characteristics on thermographic images, could indicate the presence of thyroid nodule regions.

Both studies support the possible applicability of temperature in the identification of thyroid nodules. However there are frailty: (1) patients with considerable fat layer in the neck area are not candidate to nodular detection by Infrared Thermography examinations: such examination is not indicated for obese and even overweight people; (2) the influence of the trachea, carotid arteries and jugular veins was not considered in the numerical simulation conducted by the research (study 1): they must be considered in future because such structures could be related to temperatures in the cervical region; and (3) in the CNN (study 2) the number of sample used must be increased in future works to support more grounded conclusions.

Finally, before any consideration on the use of Infrared Thermography for TN investigation, it is important a proper evaluation of the neck adipose tissue layer of the patient: thickness measurements can be done by using the same US examination recommended for nodule size evaluation, for instance.
